# Systemic inflammatory indexes mediate cardiac injury following intracerebral hemorrhage

**DOI:** 10.3389/fcvm.2026.1798009

**Published:** 2026-05-20

**Authors:** Tao Ran, Gui Li, Tian-Tian Wang, Jin-Yao Chen, Min Mao, Wen-Song Yang, Zhong Zuo

**Affiliations:** 1 Department of Cardiovascular Medicine, Cardiovascular Research Center, The First Affiliated Hospital of Chongqing Medical University, Chongqing, China; 2 Chongqing Medical University, Chongqing, China; 3 Departments of Neurology, The First Affiliated Hospital of Chongqing Medical University, Chongqing, China

**Keywords:** brain-heart axis, cardiac injury, inflammatory indices, intracerebral hemorrhage, systemic inflammation

## Abstract

**Background:**

Cardiac injury is a common complication following intracerebral hemorrhage (ICH), contributing to poor prognosis. Systemic inflammation has been proposed as a critical mediator along the brain-heart axis, linking neurological injury to remote cardiac damage. This study aimed to investigate the association between a composite inflammatory indicator and acute cardiac injury following ICH.

**Methods:**

This single-center retrospective cohort study included patients with ICH admitted within 24 h of symptom onset between 2017 and 2022. Composite inflammatory indices (*N*LR, PLR, MLR, SII, SIRI, AISI) were derived from admission peripheral blood cell counts. The primary outcome, acute cardiac injury, was defined as a hs-cTnT level >0.014 ng/mL accompanied by at least one electrocardiographic abnormality. Independent associations were evaluated using multivariable logistic regression, with dose-response relationships assessed via trend tests. Mediation analyses were conducted using PROCESS macro (models 4 and 6). Robustness was examined through stratified and sensitivity analyses.

**Results:**

Acute cardiac injury occurred in 166 (29.5%) of the 562 patients. The injury group showed significantly elevated levels of MLR, SIRI, and AISI compared to the non-injury group, each of which independently associated with cardiac injury after multivariable adjustment. These inflammatory indices partially mediated the link between lower Glasgow Coma Scale (GCS) scores and cardiac injury. A significant chain mediation pathway was identified: lower GCS → elevated SIRI → increased hs-cTnT → higher 90-day mortality. The findings were robust in stratified and sensitivity analyses.

**Conclusion:**

MLR, SIRI, and AISI serve as independent factors of acute cardiac injury after intracerebral hemorrhage and play a significant mediating role in the brain-heart axis.

## Introduction

Spontaneous intracerebral hemorrhage (ICH) carries a high burden of morbidity and mortality (1). Although neurocritical care and surgical techniques have advanced, patient outcomes remain poor. This is partly attributable to complex systemic sequelae, with cardiac complications being a key contributing factor (2, 3).

The brain-heart axis has attracted growing interest (4). Approximately 20%–30% of patients with acute brain injury experience concomitant cardiac injury, characterized by elevated cardiac enzymes, electrocardiographic abnormalities, and wall motion disturbances (5, 6). These alterations are significantly linked to worse short- and long-term prognosis (7). Current evidence indicates that cardiac injury after ICH mainly stems from brain-heart axis activation, wherein acute brain injury triggers sympathetic overdrive and a catecholamine surge, resulting in myocardial stunning and injury (8). However, emerging evidence underscores a critical role for systemic inflammation in this process (9, 10). After ICH, hematoma components and damaged brain tissue rapidly activate the innate immune system, provoking a pronounced systemic inflammatory response (11, 12). This response not only aggravates secondary brain injury but may also remotely impair cardiac function via direct myocardial toxicity or by promoting coronary plaque instability (13). Nonetheless, the role of inflammation as a bridge linking brain injury to cardiac events following ICH remains inadequately explored.

In recent years, composite inflammatory indices derived from routine complete blood counts have garnered attention due to their accessibility, low cost, and capacity to reflect systemic immune-inflammatory balance. Compared with individual cell counts, composite indices, the neutrophil-to-lymphocyte ratio (NLR) and the platelet-to-lymphocyte ratio (PLR), provide a more integrated assessment of inflammatory and immune dysregulation. The monocyte-to-lymphocyte ratio (MLR) may more specifically represent the balance between pro-inflammatory monocytes and adaptive immunity (14), while more complex markers like the systemic inflammation response index (SIRI) and the aggregate index of systemic inflammation (AISI), which incorporate neutrophils, monocytes, lymphocytes, and platelets, may better quantify the overall inflammatory burden (15). Although these indices show prognostic potential in ICH, most studies have focused only on their association with clinical outcomes, without clarifying their role in specific pathophysiological pathways. Notably, their relationship with cardiac injury within the context of brain-heart axis following ICH remains unclear. Whether they serve as key intermediaries linking severe brain injury to remote cardiac damage warrants further investigation.

Therefore, this study aims to systematically evaluate whether composite inflammatory indices mediate the association between neurological severity and acute cardiac injury after ICH, and examine their role in the pathophysiology linking brain injury to mortality.

## Materials and methods

### Standard protocol approvals, registrations, and patient consents

The study protocol was approved by the Institutional Review Board of the First Affiliated Hospital of Chongqing Medical University (Approval No. 2017-075). The study was conducted in accordance with the Declaration of Helsinki and relevant institutional guidelines. Given the retrospective design and use of de-identified data, the requirement for written informed consent was waived by the ethics committee.

### Study design and participants

This single-center retrospective cohort study utilized data from the prospective spontaneous intracerebral hemorrhage registry at the First Affiliated Hospital of Chongqing Medical University. Patients with acute spontaneous ICH admitted within 24 h of onset were consecutively enrolled from November 2017 to April 2022. Inclusion criteria were: (1) age ≥18 years; (2) hospitalization within 24 h of symptom onset; (3) radiographic confirmation of spontaneous ICH according to ICD-11 criteria. Exclusion criteria included: (1) primary intraventricular hemorrhage; (2) hemorrhagic transformation of cerebral infarction or secondary ICH (e.g., trauma, tumor, leukemia, vascular malformation); (3) multiple concurrent ICHs; (4) missing key baseline data (ECG, troponin, complete blood count); (5) lost to follow-up or unavailable 90-day outcome data.

### Clinical data collection

Two trained neurologists independently collected and verified patient data using a standardized form. Variables included demographic characteristics, risk factors, medical history, prior use of antiplatelet or anticoagulant agents, time from onset to admission, admission systolic and diastolic blood pressure, Glasgow Coma Scale (GCS) score, treatment strategy, and in-hospital complications.

Laboratory parameters included white blood cell count with differential (neutrophils, lymphocytes, monocytes), platelet count, high-sensitivity troponin T (hs-cTnT), and other routine biochemical markers. All tests were performed by the hospital laboratory following standardized protocols. Electrocardiograms were interpreted by cardiologists, with rhythm and waveform abnormalities recorded.

All patients underwent standardized non-contrast computed tomography (slice thickness: 5 mm). Admission CT images were stored in DICOM format for subsequent analysis. Imaging features were independently assessed by two experienced neuroradiologists blinded to clinical information. Hematoma volume and regions of interest (ROI) were measured using 3D Slicer software (version 5.2.2; https://www.slicer.org) via semi-automated segmentation. Discrepancies between readers were resolved by a senior physician.

### Study variables and outcome definitions

The following composite immune-inflammatory indices were calculated from peripheral blood cell counts obtained within 24 h of admission: NLR, platelet-to-lymphocyte ratio (PLR), MLR, systemic immune-inflammation index (SII), SIRI, and AISI (see Supplementary Table 1). Due to skewed distributions, all indices underwent a natural logarithm (ln) transformation before statistical analysis.

The primary outcome was acute cardiac injury, defined as hs-cTnT level >0.014 ng/mL at admission, accompanied by at least one electrocardiographic abnormality, including T-wave changes, ST-segment changes, ST-T changes, QTc prolongation, atrial fibrillation, premature beats, sinus tachycardia, significant sinus bradycardia defined as a heart rate below 50 beats per minute, atrioventricular blocks, and bundle branch blocks. The secondary outcome was 90-day all-cause mortality, ascertained through outpatient follow-up or telephone interview.

### Statistical analysis

All analyses were performed using R (v4.4.3) and SPSS (v27.0). Continuous variables were summarized as mean ± SD or median (IQR) and compared using *t*-tests or Mann–Whitney *U*-tests, as appropriate. Categorical variables were reported as frequency (%) and compared using chi-square or Fisher's exact tests.

Multivariable logistic regression was used to assess independent associations between inflammatory indices and acute cardiac injury. Dose-response relationships were evaluated by modeling indices as quartiles and calculating *P* for trend. Mediation analyses were conducted using the PROCESS macro: Model 4 tested the simple mediation effect of inflammatory indices on the GCS-cardiac injury relationship, and Model 6 examined the chain mediation pathway “GCS → inflammatory index → hs-cTnT → 90-day mortality.” Indirect effects were estimated with bias-corrected 95% CIs from 5000 bootstrap samples. Stratified analyses by ICH severity and sensitivity analyses excluding patients with GCS ≤ 8 were performed to verify robustness. To assess the discriminative performance of inflammatory indices for acute cardiac injury, we conducted ROC curve analysis and calculated the AUC with 95% CIs for each index. A two-sided *P* < 0.05 was considered statistically significant.

## Results

### Cohort characteristics

**Figure 1:**
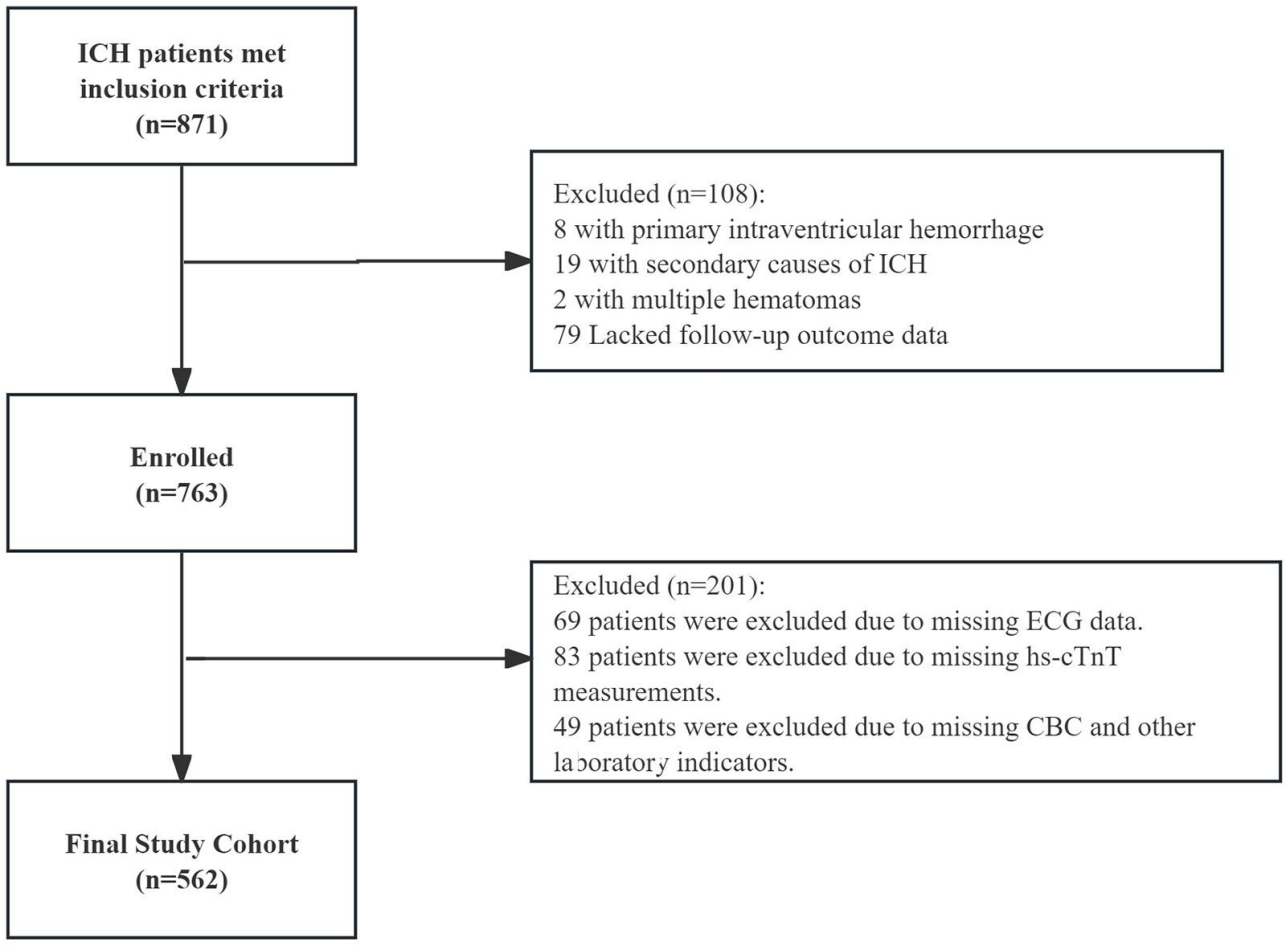
Flowchart of patient selection. From 871 eligible patients, exclusions were made for primary intraventricular hemorrhage, secondary causes, multiple hematomas, missing follow-up data, and missing key clinical or laboratory variables (ECG, hs-cTnT, WBC). A final cohort of 562 patients was included.

A total of 562 patients with ICH were included in the final analysis (Figure 1). Of these, 166 patients (29.5%) developed acute cardiac injury. Compared to patients without cardiac injury, those in the injury group were older; had a higher prevalence of antiplatelet medication use, diabetes, hypertension, and coronary heart disease; and presented with significantly higher admission SBP, larger hematoma volumes, and greater frequency of intraventricular hemorrhage (IVH). Admission GCS scores were significantly lower in the cardiac injury group.

### Inflammatory indices and neurological severity

Spearman correlation analysis was performed to assess the relationship between neurological severity and systemic inflammation. All ln-transformed inflammatory indices were significantly correlated with admission GCS score (all *p* < 0.01). SIRI showed the strongest correlation (*r* = −0.467), followed by NLR (*r* = −0.448) and AISI (*r* = −0.415). MLR (*r* = −0.328) and PLR (*r* = −0.184) showed weaker but still significant correlations. These findings indicate that greater neurological impairment is associated with a heightened systemic inflammatory response (Supplementary Table 2).

### Association of inflammatory indices with acute cardiac injury

**Figure 2:**
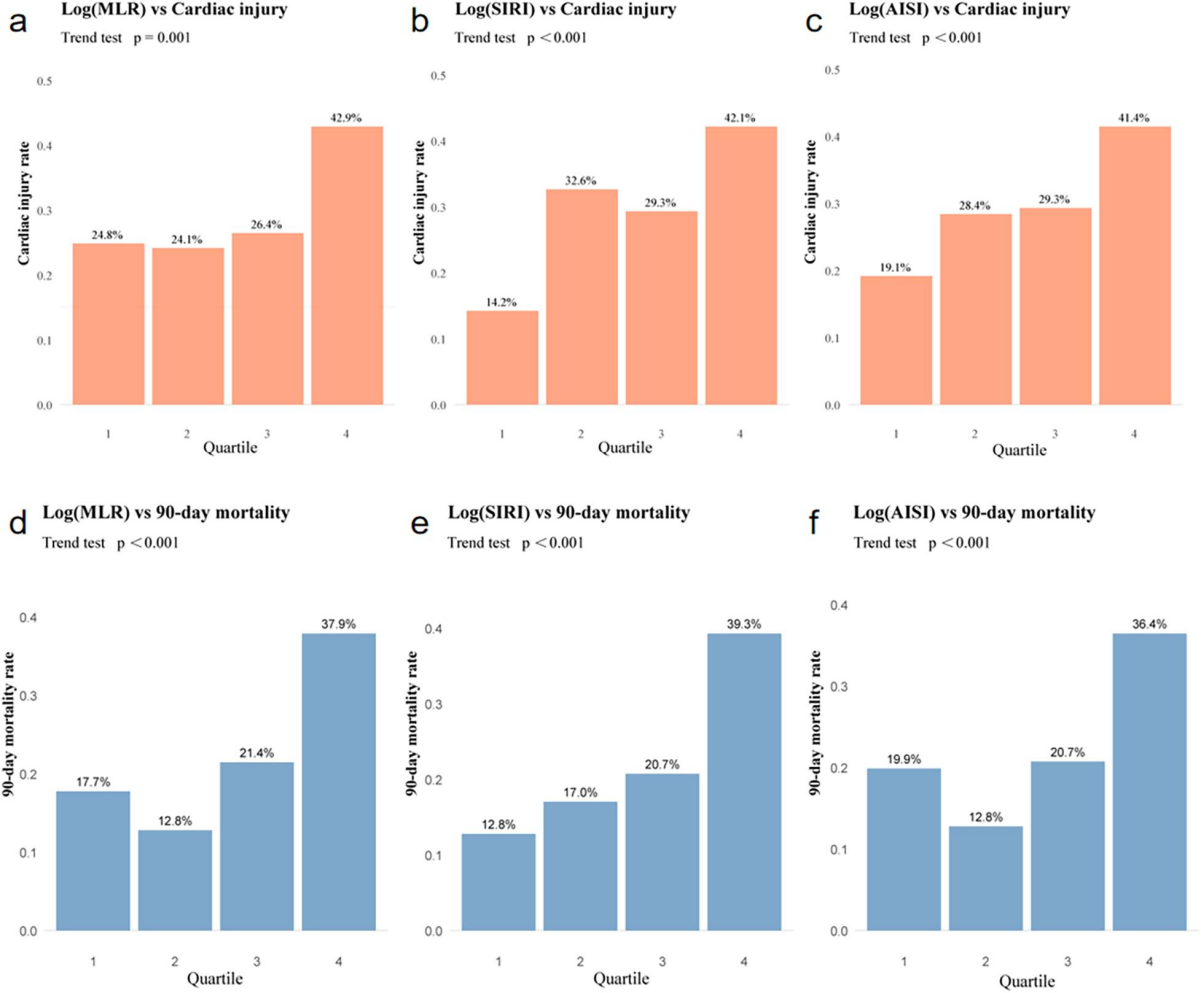
Dose-response associations of inflammatory indices with cardiac injury and 90-day mortality. **(a–c)** Increasing quartiles of Ln(MLR), Ln(SIRI), and Ln(AISI) are associated with higher rates of acute cardiac injury (*P* for trend <0.001). **(d–f)** Corresponding associations with 90-day all-cause mortality (*P* for trend <0.001). Panels a-c show progressively increased rates of cardiac injury across quartiles of Log(MLR), Log(SIRI), and Log(AISI), respectively (all trend *p* < 0.001). Panels **(d–f)** display the corresponding associations between these inflammatory markers and 90-day mortality, with higher quartiles consistently showing elevated mortality rates (all trend *p* < 0.001). Numerical values above each bar represent observed event proportions.

Most composite inflammatory indices, including NLR, MLR, SII, SIRI, and AISI, were significantly elevated in patients with cardiac injury compared to those without (Table 1). After adjusting for potential confounders, multivariable logistic regression showed that MLR, SIRI, and AISI remained independently associated with cardiac injury. The corresponding odds ratios (ORs) are presented in Table 2. In contrast, NLR, PLR, and SII were no longer significant after adjustment (Table 2). Quartile-based trend analysis further demonstrated significant dose-response relationships for MLR, SIRI, and AISI, with progressively higher cardiac injury rates across increasing quartiles (*P* for trend < 0.05) (Figure 2). These findings suggest that a greater systemic inflammatory burden is associated with an increased risk of acute cardiac injury.

**Table 1:** Baseline characteristics of ICH patients, stratified by the presence of acute cardiac injury.

Variables	Patients without cardiac injury (*n* = 396, 70.5%)	Patients with cardiac injury (*n* = 166, 29.5%)	*p* value
Demographic			
Age, y (SD)	61.8 (12.3)	63.2 (16.3)	0.323
Sex, male, *n* (%)	286 (72.2)	121 (72.9)	0.871
Smoking, *n* (%)	185 (46.7)	74 (44.6)	0.643
Alcohol consumption, *n* (%)	127 (32.1)	54 (32.5)	0.915
Anticoagulant use before ICH onset, *n* (%)	6 (1.5)	4 (2.4)	0.492
Antiplatelet use before ICH onset, *n* (%)	24 (6.1)	21 (12.7)	**0** **.** **009**
Diabetes mellitus, *n* (%)	42 (10.6)	33 (19.9)	**0** **.** **003**
History of Hypertension, *n* (%)	215 (54.3)	113 (68.1)	**0** **.** **003**
History of coronary heart disease, *n* (%)	18 (4.5)	16 (9.6)	**0** **.** **021**
Clinical characteristics			
Admission SBP, mmHg (SD)	165.5 (25.8)	174.4 (32.7)	**0** **.** **002**
Admission DBP, mmHg (SD)	95.7 (17.5)	97.3 (23.5)	0.411
Admission GCS score, (IQR)	14.0 [11.0,15.0]	12.0 [7.0,14.0]	**<0** **.** **001**
Imaging features			
Time from onset to CT, h (IQR)	4.0 [2.0,7.2]	3.0 [1.5,7.0]	0.290
Midline shift, mm (IQR)	3.0 [0.0,5.1]	4.1 [0.0,8.6]	**0** **.** **003**
Baseline ICH volume, mL (IQR)	12.2 [4.7,26.7]	18.0 [7.7,51.6]	**<0** **.** **001**
IVH, *n* (%)	138 (34.8)	93 (56.0)	**<0** **.** **001**
Hematoma location			
Cerebellum, *n* (%)	16 (4.7)	8 (5.2)	0.992
Thalamus, *n* (%)	82 (24.0)	35 (22.6)	
Brainstem, *n* (%)	29 (8.5)	14 (9.0)	
Basal ganglia, *n* (%)	146 (42.7)	65 (41.9)	
Lobar region, *n* (%)	69 (20.2)	33 (21.3)	
Composite immune-inflammatory indices			
NLR, (IQR)	6.8 [3.9, 11.9]	9.1 [5.2, 15.2]	**<0** **.** **001**
PLR, (IQR)	169.4 [121.9, 240.0]	185.9 [124.2, 284.1]	0.286
MLR, (IQR)	0.4 [0.3, 0.6]	0.5 [0.3, 0.8]	**<0** **.** **001**
SII, (IQR)	1,170.2 [681.7, 2,177.9]	1,615.0 [895.5,2, 779.9]	**0** **.** **002**
SIRI, (IQR)	2.5 [1.3, 5.2]	3.9 [2.3, 8.0]	**<0** **.** **001**
AISI, (IQR)	469.2 [221.0, 907.5]	634.0 [337.5, 1,465.4]	**<0** **.** **001**

Bold values indicate statistical significance (*p* < 0.05).

**Table 2:** Multivariable logistic regression analysis of inflammatory markers associated with cardiac injury.

Inflammatory marker	Coefficient (B)	*P*-value	Odds ratio	95% confidence interval
Log(NLR)	0.10	0.769	1.10	0.59–2.07
Log(PLR)	−0.19	0.646	0.83	0.38–1.83
Log(MLR)	0.72	**0.049**	2.05	1.00–4.18
Log(SII)	0.12	0.688	1.12	0.64–1.99
Log(SIRI)	0.56	**0.034**	1.75	1.04–2.92
Log(AISI)	0.48	**0.042**	1.62	1.02–2.58

Data are presented as mean ± SD, median [IQR], or *n* (%). ICH, intracerebral hemorrhage; IVH, intraventricular hemorrhage; SBP, systolic blood pressure; DBP, diastolic blood pressure; GCS, Glasgow Coma Scale.

Bold values indicate statistical significance (*p* < 0.05).

### Mediation analysis

**Figure 3:**
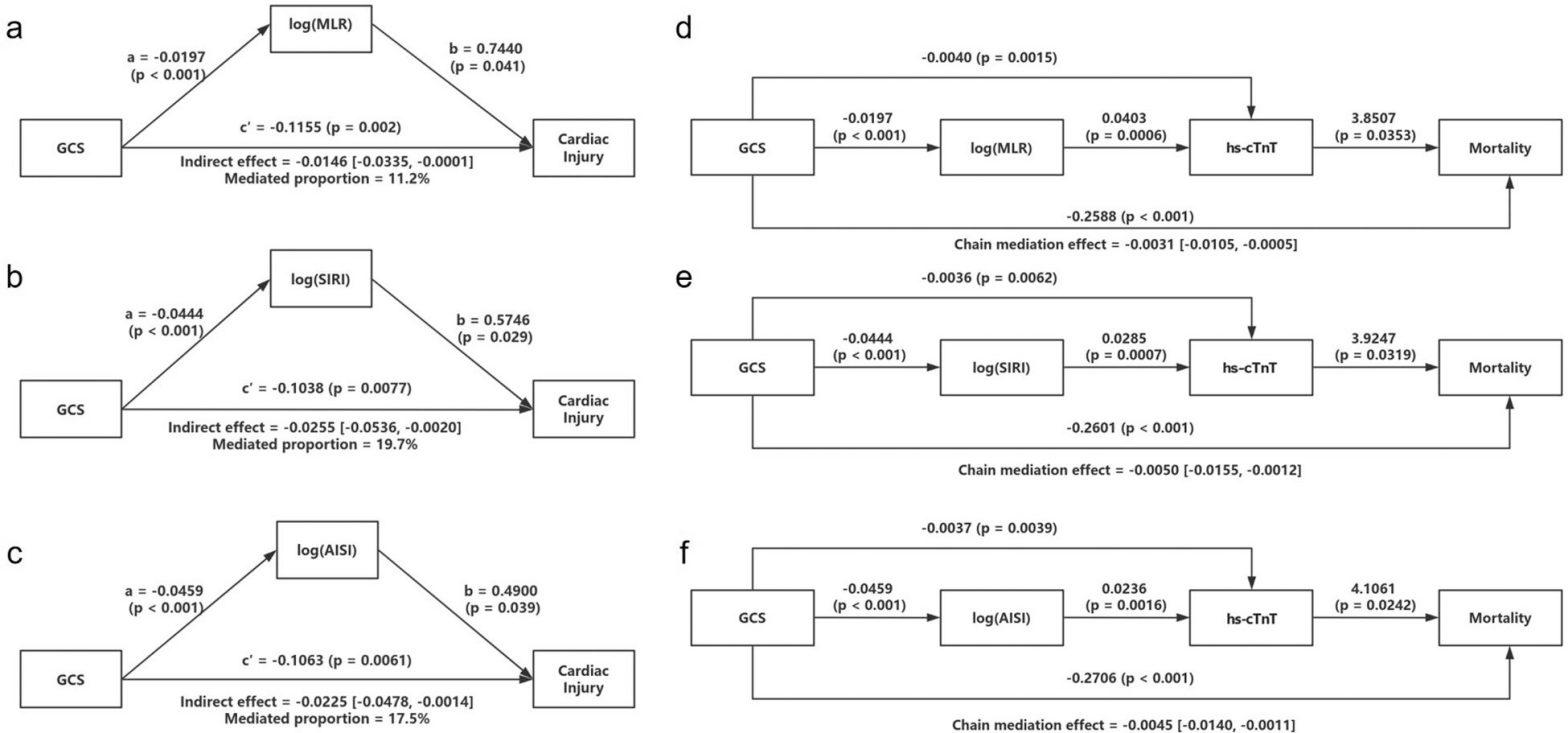
Mediation and sequential mediation analyses of the associations between GCS, inflammatory markers, cardiac injury, and mortality. Mediation models (panels **a–c**) and sequential mediation models (panels **d–f**) illustrating the indirect effects of Log(MLR), Log(SIRI), and Log(AISI) on the associations between admission GCS and cardiac injury or 90-day mortality. Panels a-c depict single-mediator models showing that Log(MLR), Log(SIRI), and Log(AISI) partially mediated the associations between GCS and cardiac injury. Path coefficients (**a, b, and c′**), indirect effects with 95% confidence intervals, and mediated proportions are presented for each model. Panels **d–f** depict sequential mediation models in which inflammatory markers (M1) and hs-cTnT (M2) jointly mediated the association between GCS and 90-day mortality. Significant chained indirect effects (a1 × d21 × b2) were observed across all three inflammatory markers, with the corresponding 95% confidence intervals shown below each diagram.

Given the significant associations between GCS and inflammatory indices, as well as between inflammatory indices and cardiac injury, we next examined whether inflammation mediated the relationship between neurological severity and cardiac injury (Figure 3). Simple mediation analysis revealed that GCS partly influenced cardiac injury risk through elevated MLR, SIRI, and AISI levels, with statistically significant indirect effects for all three indices (bootstrap 95% CIs excluding zero) (Table 3). The proportion of the total effect mediated by these indices ranged from 10.4% for MLR to 19.7% for SIRI. Notably, SIRI demonstrated the highest mediation proportion (19.7%), suggesting that systemic inflammation accounts for approximately one-fifth of the total effect of neurological impairment on cardiac injury, highlighting its stronger role in the “neurological impairment → cardiac injury” pathway.

**Table 3:** Simple mediation analysis of inflammatory markers in the association between GCS and cardiac injury.

Inflammatory marker	Path a: GCS → marker *β* (*p*-value)	Path b: marker → cardiac injury *β* (*p*-value)	Direct effect c′ *β* (*p*-value)	Indirect effect	95% CI (Bootstrap)	Mediation proportion (%)
MLR	−0.0197 (*p* < 0.001)	0.7440 (*p* = 0.041)	−0.1155 (*p* = 0.002)	−0.0146	−0.0335, −0.0001	11.20%
SIRI	−0.0444 (*p* < 0.001)	0.5746 (*p* = 0.029)	−0.1038 (*p* = 0.0077)	−0.0255	−0.0536, −0.0020	19.70%
AISI	−0.0459 (*p* < 0.001)	0.4900 (*p* = 0.039)	−0.1063 (*p* = 0.0061)	−0.0225	−0.0478, −0.0014	17.50%

All models were adjusted for age, intraventricular hemorrhage, hypertension, diabetes mellitus, hematoma volume, and systolic blood pressure. Path a represents the effect of GCS on inflammatory markers; Path b represents the effect of inflammatory markers on cardiac injury; Direct effect c′ represents the direct effect of GCS on cardiac injury after accounting for the mediation pathway. Bootstrap confidence intervals were calculated with 5,000 resamples. All mediation effects were statistically significant (*p* < 0.05).

Chain mediation analysis further revealed that GCS affected 90-day mortality not only through the inflammatory pathway but also via inflammation-driven hs-cTnT elevation. Significant chain mediation effects were observed for MLR, SIRI, and AISI (Table 4), with SIRI showing the most prominent effect. These findings indicate that the pathway from neurological severity to mortality involves a sequential cascade: neurological impairment leads to systemic inflammation, which in turn causes myocardial injury (reflected by hs-cTnT elevation), ultimately contributing to increased mortality. The consistent results across all three indices reinforce the robustness of this chain mediation pathway and underscore the potential importance of the “inflammation → myocardial injury → mortality” cascade. A schematic representation of the proposed sequential mediation pathway is shown in Supplementary Figure 1.

**Table 4:** Chain mediation analysis of inflammatory markers and cardiac injury in the association between GCS and mortality.

Inflammatory marker	Path a1: GCS → M1 β (*p*-value)	Path d21: M1 → hs-cTnT β (*p*-value)	Path a2: GCS → hs-cTnT β (*p*-value)	Path b2: hs-cTnT → mortality β (*p*-value)	Direct effect c′: GCS → mortality β (*p*-value)	Chain mediation effect (95% CI)
MLR	−0.0197 (*p* < 0.001)	0.0403 (*p* = 0.0006)	−0.0040 (*p* = 0.0015)	3.8507 (*p* = 0.0353)	−0.2588 (*p* < 0.001)	−0.0031 (−0.0105, −0.0005)
SIRI	−0.0444 (*p* < 0.001)	0.0285 (*p* = 0.0007)	−0.0036 (*p* = 0.0062)	3.9247 (*p* = 0.0319)	−0.2601 (*p* < 0.001)	−0.0050 (−0.0155, −0.0012)
AISI	−0.0459 (*p* < 0.001)	0.0236 (*p* = 0.0016)	−0.0037 (*p* = 0.0039)	4.1061 (*p* = 0.0242)	−0.2706 (*p* < 0.001)	−0.0045 (−0.0140, −0.0011)

All models were adjusted for age, intraventricular hemorrhage, hypertension, diabetes mellitus, hematoma volume, and systolic blood pressure. Path a1 represents the effect of GCS on inflammatory markers; Path d21 represents the effect of inflammatory markers on cardiac injury (hs-cTnT); Path a2 represents the direct effect of GCS on cardiac injury; Path b2 represents the effect of cardiac injury on mortality; Direct effect c′ represents the direct effect of GCS on mortality after accounting for the chain mediation pathway. All chain mediation effects were statistically significant.

### Subgroup and sensitivity analyses

To further explore whether the observed associations between inflammatory indices and cardiac injury were confounded by overall disease severity, we performed stratified analyses by ICH severity. As shown in Supplementary Table 3, the associations varied across inflammatory markers. MLR was significantly associated with cardiac injury only in the mild group (OR: 4.96, 95% CI: 1.54–15.95), whereas SIRI showed significant associations in both the mild and moderate-to-severe groups. AISI was significantly associated in the moderate-to-severe group (OR: 1.95, 95% CI: 1.14–3.34) and showed a borderline association in the mild group (OR: 1.90, 95% CI: 0.96–3.75). These findings suggest that the role of inflammation in cardiac injury is not merely a reflection of disease severity, as significant associations persisted even in patients with milder neurological impairment. To assess whether the results were driven by the most critically ill patients, we performed a sensitivity analysis excluding patients with severe neurological impairment (GCS ≤ 8). After exclusion, the associations between MLR, SIRI, and AISI and cardiac injury remained robust, with effect sizes and statistical significance consistent with the primary analysis (Supplementary Table 4). This further supports the robustness of our findings and suggests that the observed associations are not solely attributable to the most severe cases.

### Predictive performance of inflammatory indices for acute cardiac injury

To evaluate the discriminative ability of each inflammatory index for identifying patients at risk of acute cardiac injury, we performed receiver operating characteristic (ROC) curve analysis. As shown in Supplementary Table 5, the area under the curve (AUC) values ranged from 0.529 to 0.629. SIRI achieved the highest AUC (0.629, 95% CI: 0.579–0.678), followed by AISI (0.610, 95% CI: 0.560–0.660) and MLR (0.602, 95% CI: 0.549–0.655). These AUC values indicate that none of the inflammatory indices alone have good discriminative power (all <0.7), suggesting that they are not suitable as standalone screening or diagnostic tools for acute cardiac injury in this setting.

## Discussion

This study demonstrates that composite inflammatory indices, particularly MLR, SIRI, and AISI, serve as independent factors of acute cardiac injury following spontaneous intracerebral hemorrhage. Furthermore, these indices play a key mediating role in the brain-heart axis, partially mediating the effect of brain injury on 90-day mortality.

ICH triggers a robust immune-inflammatory response. Hematoma formation and blood-brain barrier disruption lead to neutrophil and monocyte infiltration into the central nervous system, accompanied by a cascade of pro-inflammatory cytokines that exacerbate secondary brain injury (16). This systemic inflammation extends beyond the brain and can impair peripheral organs, particularly the heart, via autonomic and humoral pathways (17). Acute stroke may induce sympathetic overactivation and increased inflammatory mediators, resulting in stress-induced myocardial injury, termed the brain-heart axis effect (18). Elevated high-sensitivity C-reactive protein is independently associated with increased high-sensitivity troponin T, indicating a higher risk of myocardial injury in patients with greater inflammatory burden (19).

Among the composite inflammatory indices evaluated, MLR, SIRI, and AISI, but not NLR or PLR, remained independently associated with cardiac injury after multivariable adjustment. This differential predictive capacity may be explained by their distinct biological underpinnings. NLR and PLR primarily reflect acute neutrophilic inflammation and platelet activity, respectively, whereas MLR incorporates monocytes, which are central to sustained inflammation and tissue repair processes. SIRI and AISI further integrate multiple leukocyte lineages, potentially offering a more comprehensive quantification of the systemic inflammatory burden that drives remote myocardial injury. This suggests that monocyte-lymphocyte imbalance and systemic inflammatory load are key contributors to cardiac injury via the brain-heart axis. Monocyte-driven inflammation tends to persist and is involved in tissue repair and fibrosis, making it more reflective of myocardial involvement (20). Prior studies support this, showing that SIRI, which includes neutrophil and monocyte counts, outperforms NLR in predicting ICH outcomes (21). Additionally, absolute monocyte count has been identified as an independent risk factor for early mortality in ICH (21). AISI integrates neutrophils, monocytes, platelets, and lymphocytes, offering a more comprehensive measure of systemic inflammation (22). The significance of AISI in our findings further highlights the role of multi-cellular inflammation in ICH-related myocardial injury. These indices (MLR, SIRI, AISI) likely offer superior sensitivity by capturing the extent and complexity of the immune-inflammatory response, beyond isolated cell changes. While these indices likely offer a more comprehensive reflection of the systemic inflammatory response than isolated cell counts, their modest individual discriminative power, as indicated by the ROC analysis, suggests they should be viewed as markers of biological risk rather than highly sensitive standalone diagnostic tools.

Disease severity may affect the association between inflammatory indices and cardiac injury (23). Importantly, these indices may serve as true biological mediators along the brain-heart axis or simply reflect overall disease severity. Distinguishing between these two roles is critical for mechanistic interpretation. Previous studies have shown that patients with large hematomas or critical conditions often have high baseline mortality and intense stress responses, which may weaken the marginal effects of inflammatory indices (24). Moreover, severe patients exhibit high short-term mortality regardless of cardiac injury (25). These factors suggest that, in unstratified analyses, strong inflammation in severe cases may mask true associations. Therefore, we performed stratified analyses by ICH severity based on admission GCS score to evaluate the associations between inflammatory indices and cardiac injury across different severity subgroups. The stratified analyses revealed that the associations varied across inflammatory markers. MLR was significantly associated with cardiac injury only in the mild group (GCS: 13–15), whereas SIRI showed significant associations in both the mild and moderate-to-severe groups, and AISI was significantly associated in the moderate-to-severe group (GCS: 3–12). Since confounding by disease severity is less pronounced in the mild subgroup, the persistence of significant associations in this group suggests that the role of inflammation extends beyond merely reflecting overall disease severity. However, residual confounding cannot be fully excluded, and mediation analysis based on observational data does not establish causality. Prospective studies are needed to confirm inflammation as a true biological mediator in the brain-heart axis.

Our mediation and chain mediation analyses quantitatively assessed the role of systemic inflammation in ICH-induced cardiac injury. Results showed that inflammatory responses partially mediate both acute cardiac injury and long-term mortality after ICH, supporting the brain-heart axis pathophysiology. Acute brain injury triggers catecholamine surges and systemic inflammation, leading to myocardial damage via *β*-adrenergic overstimulation and microcirculatory dysfunction (26, 27). These findings provide clinical evidence for this mechanism, showing that elevated inflammatory indices are associated with increased risks of myocardial injury and mortality. However, mediation analyses based on observational data are subject to unmeasured confounding, and further prospective studies are needed to validate the proposed mechanisms.

Our findings align with existing stroke research. Multiple studies have reported a high incidence of myocardial injury after acute stroke, strongly linked to poor outcomes (28, 29). This study extends prior work by evaluating accessible peripheral inflammatory markers in relation to cardiac injury after ICH. These indices are widely available, easy to measure, and cost-effective. However, the exploratory ROC analysis showed only limited discriminative performance when these indices were used alone. Therefore, MLR, SIRI, and AISI should not be interpreted as stand-alone diagnostic or predictive tools, but rather as potential markers of inflammatory burden that may complement clinical assessment and future multivariable risk models. Unlike previous studies focusing mainly on neurological outcomes, our research broadens the risk assessment framework through the brain-heart axis, suggesting that inflammatory burden may link brain injury to myocardial damage. This view may provide a useful framework for further investigating systemic complications after ICH. Future research and interventions should address neurological and cardiac complications as part of an integrated brain-heart axis pathway, with inflammation as a central mediator. Targeting inflammatory signaling may disrupt the cycle of brain injury, systemic stress, and myocardial damage, improving both neurological and cardiac outcomes in ICH (30).

## Limitations

This study has several limitations. First, as a single-center retrospective analysis with a limited sample size, selection bias may be present, and the generalizability of our findings requires validation in multi-center studies. Second, key physiological factors such as autonomic function and stress hormones were not assessed; these unmeasured variables may influence post-ICH cardiac injury. Finally, as the mediation analysis relied on cross-sectional data, causal inference is inherently limited.

## Conclusion

In ICH, MLR, SIRI, and AISI are independently associated with acute cardiac injury, although their standalone predictive performance is modest. Our findings suggest that systemic inflammation may serve as a potential pathway within the brain-heart axis, partially linking neurological impairment to patient prognosis. These preliminary results provide a basis for further exploring inflammatory markers in risk stratification and management.

## Data Availability

The data analyzed in this study is subject to the following licenses/restrictions: Anonymized data not published within this article will be made available by request from any qualified investigator. Requests to access these datasets should be directed to Wen-Song Yang, yangwensong@hospital.cqmu.edu.cn.
