# Supplementary material for: Systemic inflammatory indexes mediate cardiac injury following intracerebral hemorrhage
Source: Front Cardiovasc Med. 2026 May 20;13:1798009. doi: 10.3389/fcvm.2026.1798009 (PMC13229705; doi:10.3389/fcvm.2026.1798009)
Supplement: Supplementary file 1 [file Datasheet1.docx]

Supplementary Material

# Supplementary Tables

**Supplementary Table 1.** Supplementary Table 1. Definitions, formulae, and interpretations of composite inflammatory indices derived from peripheral blood cell counts

| Abbreviation | Full Name | Formula | Putative Biological Interpretation |
| --- | --- | --- | --- |
| **NLR** | Neutrophil-to-Lymphocyte Ratio | Neutrophil Count / Lymphocyte Count | Reflects the balance between the innate immune/acute inflammatory response (neutrophils) and adaptive immune regulation (lymphocytes). A higher ratio indicates predominant inflammation and relative immune suppression. |
| **PLR** | Platelet-to-Lymphocyte Ratio | Platelet Count / Lymphocyte Count | Integrates information about thrombosis potential (platelets) and immune status (lymphocytes). It may represent a link between inflammatory states and pro-thrombotic tendencies. |
| **MLR** | Monocyte-to-Lymphocyte Ratio | Monocyte Count / Lymphocyte Count | Represents the equilibrium between innate immunity/inflammatory chronicity (monocytes) and adaptive immune competence (lymphocytes). It is thought to be particularly sensitive to sustained inflammatory burden. |
| **SII** | Systemic Immune-Inflammation Index | (Neutrophil Count × Platelet Count) / Lymphocyte Count | A comprehensive index that simultaneously quantifies the status of innate immunity (neutrophils), thrombosis/inflammation (platelets), and adaptive immunity (lymphocytes). It is considered a strong marker of systemic inflammatory burden. |
| **SIRI** | Systemic Inflammation Response Index | (Neutrophil Count × Monocyte Count) / Lymphocyte Count | Integrates key cellular mediators of the innate immune response (neutrophils and monocytes) relative to adaptive immunity (lymphocytes). It may provide a nuanced assessment of the active systemic inflammatory response. |
| **AISI** | Aggregate Index of Systemic Inflammation | (Neutrophil Count × Monocyte Count × Platelet Count) / Lymphocyte Count | The most complex index, aggregating three pro-inflammatory/pro-thrombotic cell lines (neutrophils, monocytes, platelets) against the adaptive immune compartment (lymphocytes). It is hypothesized to represent the overall "volume" of systemic inflammation. |

**All cell counts are expressed as ×10⁹/L. Indices were natural log-transformed for analysis due to skewed distributions.**

**Supplementary Table 2.**Spearman correlation matrix of inflammatory indices and GCS

| **Variable** | **r** | **P value** |
| --- | --- | --- |
| NLR | -0.448 | <0.001 |
| PLR | -0.184 | <0.001 |
| MLR | -0.328 | <0.001 |
| SII | -0.397 | <0.001 |
| SIRI | -0.467 | <0.001 |
| AISI | -0.415 | <0.001 |

## **Supplementary Table 3.** Stratified Analysis of Inflammatory Markers and Cardiac Injury by Intracerebral Hemorrhage Severity

| **Inflammatory Marker** | **ICH Severity** | **Sample Size** | **Cardiac Injury Cases** | **Cardiac Injury Rate (%)** | **OR (95% CI)** | **P-value** |
| --- | --- | --- | --- | --- | --- | --- |
| **MLR** | Mild | 318 | 69 | 21.7 | **4.96 (1.54-15.95)** | ****0.007**** |
|  | Moderate-to-Severe | 244 | 97 | 39.8 | 2.02 (0.89-4.58) | 0.094 |
| **SIRI** | Mild | 318 | 69 | 21.7 | **3.02 (1.40-6.50)** | ****0.005**** |
|  | Moderate-to-Severe | 244 | 97 | 39.8 | **1.91 (1.06-3.45)** | ****0.032**** |
| **AISI** | Mild | 318 | 69 | 21.7 | 1.90 (0.96-3.75) | 0.066 |
|  | Moderate-to-Severe | 244 | 97 | 39.8 | **1.95 (1.14-3.34)** | ****0.015**** |

ICH severity was categorized based on admission Glasgow Coma Scale (GCS) score: mild ICH was defined as GCS 13–15, and moderate-to-severe ICH was defined as GCS 3–12.

## **Supplementary Table 4.** Sensitivity Analysis of Inflammatory Markers and Cardiac Injury After Excluding Patients with Severe Neurological Impairment

| **Inflammatory Marker** | **Analysis Type** | **Sample Size** | **Cardiac Injury Cases** | **Cardiac Injury Rate (%)** | **OR (95% CI)** | **P-value** |
| --- | --- | --- | --- | --- | --- | --- |
| **MLR** | After excluding GCS≤8 | 447 | 108 | 24.2 | **5.33 (2.25-12.63)** | ****<0.001**** |
| **SIRI** | After excluding GCS≤8 | 447 | 108 | 24.2 | **2.92 (1.66-5.14)** | ****<0.001**** |
| **AISI** | After excluding GCS≤8 | 447 | 108 | 24.2 | **2.28 (1.36-3.81)** | ****0.002**** |

## **Supplementary Table 5.** Exploratory ROC Curve Analysis of Inflammatory Indices for Identifying Acute Cardiac Injury

| **Variable** | **AUC** | **SE** | **P value** | **95% CI** |
| --- | --- | --- | --- | --- |
| NLR | 0.598 | 0.026 | <0.001 | 0.547-0.648 |
| PLR | 0.529 | 0.027 | 0.286 | 0.475-0.582 |
| MLR | 0.602 | 0.027 | <0.001 | 0.549-0.655 |
| SII | 0.584 | 0.026 | 0.002 | 0.533-0.635 |
| SIRI | 0.629 | 0.025 | <0.001 | 0.579-0.678 |
| AISI | 0.610 | 0.026 | <0.001 | 0.560-0.660 |

# Supplementary Figure

#
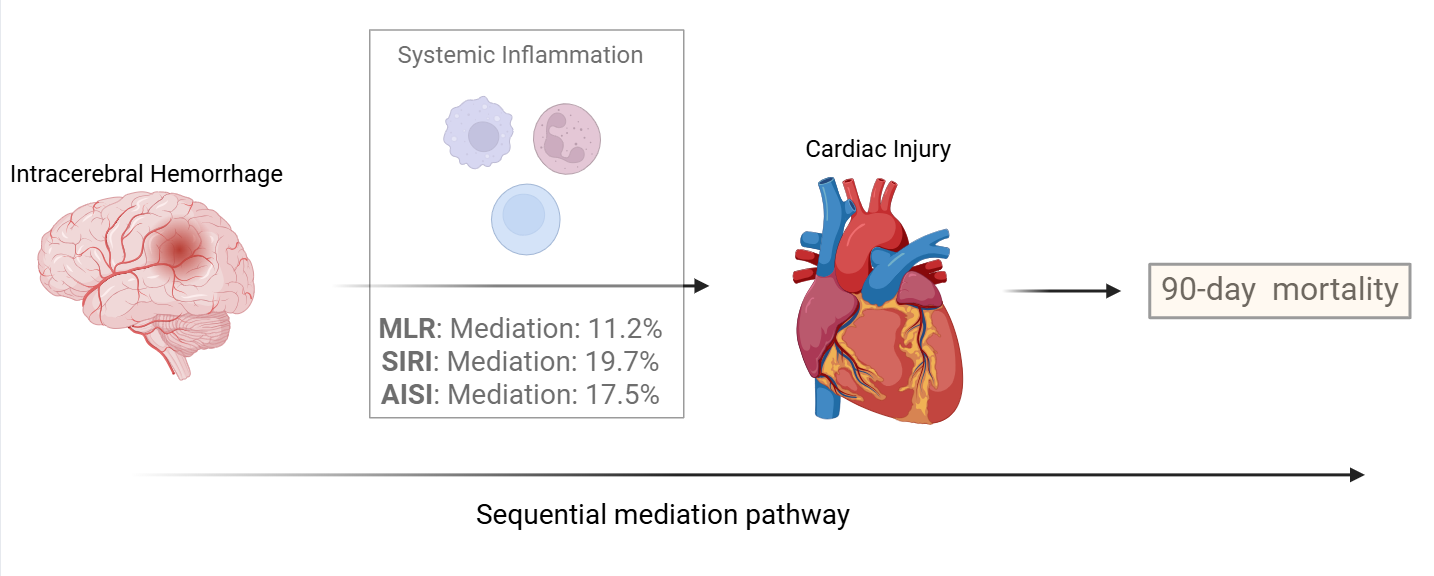


# **Supplementary Figure 1. Sequential mediation pathway among intracerebral hemorrhage, systemic inflammation, cardiac injury, and 90-day mortality.** This schematic illustrates the proposed mediation framework in which MLR, SIRI, and AISI may partly mediate the association between intracerebral hemorrhage, cardiac injury, and 90-day mortality, with mediated proportions of 11.2%, 19.7%, and 17.5%, respectively. Created with BioRender.com.
